# Healthcare Disparities in the Management of Indolent Mycosis Fungoides

**DOI:** 10.7759/cureus.24098

**Published:** 2022-04-13

**Authors:** Mădălina Laura Banciu, Elena Codruta Dobrica, Cristina Soare, Ana Maria Malciu, Vlad Mihai Voiculescu

**Affiliations:** 1 Dermatology, Elias Emergency University Hospital, Bucharest, ROU; 2 Pathophysiology, University of Medicine and Pharmacy of Craiova, Craiova, ROU; 3 Dermatology, Carol Davila University of Medicine and Pharmacy, Bucharest, ROU

**Keywords:** retinoids, large plaque parapsoriasis, chemotherapy, cutaneous t-cell lymphoma, mycosis fungoides

## Abstract

Mycosis fungoides represents the most common cutaneous T-cell lymphoma, clinically manifested with evolving skin lesions, including patches, plaques, tumors, and erythroderma. Early diagnosis remains difficult to establish because it mimics several benign skin conditions, but maintaining a high index of suspicion for the disease is essential in preventing the progression of a potentially fatal disease. We report the case of a 69-year-old female who presented in our dermatology clinic in 2018 with scaly, indurated, itchy erythematous-violaceus patches and plaques, and tumors disseminated throughout the skin evolving for nine years. Skin biopsy supplemented with immunohistochemical staining established the diagnosis of mycosis fungoides. Due to the equivocal clinical presentation and the lack of extracutaneous manifestations, the patient received conventional therapy according to the stage of the disease. The rapidly progressive evolution of the cutaneous lesions in the last year of the disease determined the patient’s death despite instituting systemic chemotherapy. Patient follow-up and a multidisciplinary approach are essential to diagnose and manage this disease in its early stages. This will prevent the progression to a life-threatening malignancy and the use of immunosuppressive therapy, which can cause serious side effects.

## Introduction

Primary cutaneous lymphomas define a group of extranodal non-Hodgkin’s lymphomas originating from T, B, or natural killer lymphocytes [1]. About 75-80% of primary cutaneous lymphomas derive from different subsets of T lymphocytes, and of these, mycosis fungoides is the most prevalent subtype, accounting for 50-65% of cases [2]. Although it is considered a rare disease, the incidence has shown an increasing trend in recent decades, probably due to improved diagnostic criteria and a rise in the population’s life expectancy [3]. Mycosis fungoides tends to affect adults aged 50 to 60, with a predominance in males and the African-American population [2].

Mycosis fungoides presents a typical evolution, with three stages: patch, plaque, and tumor. At the onset, the disease appears as erythematous patches with well-defined edges in non-photo exposed areas. These are nonspecific lesions characterized by interindividual variability because they can be hyperpigmented/hypopigmented, poikilodermic, or lichenoid. This nonspecific clinical presentation can delay the diagnosis by 3-6 years [3]. Less than 10% of patients progress to more advanced stages. Over time, the lesions expand, become well-defined, infiltrated, brown-purple plaques, with asymmetrical distribution, and some may fuse, causing arched lesional areas [1]. From this stage, 25-30% of patients progress to the tumor stage. Some patients may develop erythrodermic mycosis fungoides, characterized by erythroderma with severe itching and scaling [3]. The lesions become papular or nodular as the disease progresses, sometimes with ulcerated tumors at risk of superinfection. The risk of death increases as the disease progresses, but it exists even in the early stages [4].

Diagnosis is challenging for both the clinician and the pathologist, especially in the early stages of the disease. Biopsies can be inconclusive for a long time since the clinical manifestations mimic benign conditions such as chronic contact dermatitis, pityriasis lichenoides chronica, psoriasis, parapsoriasis, or atopic dermatitis. Therefore, the main determinants of these patients’ prognosis remain the correlation of clinical and laboratory data to establish the diagnosis and the early initiation of treatment [5].

## Case presentation

**Figure 1:**
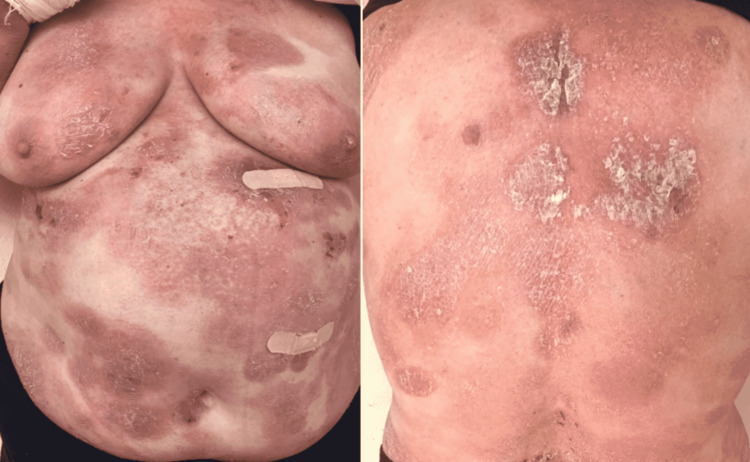
Erythematous-violaceus, scaly, indurated, plaques, and patches disseminated throughout the skin

**Figure 2:**
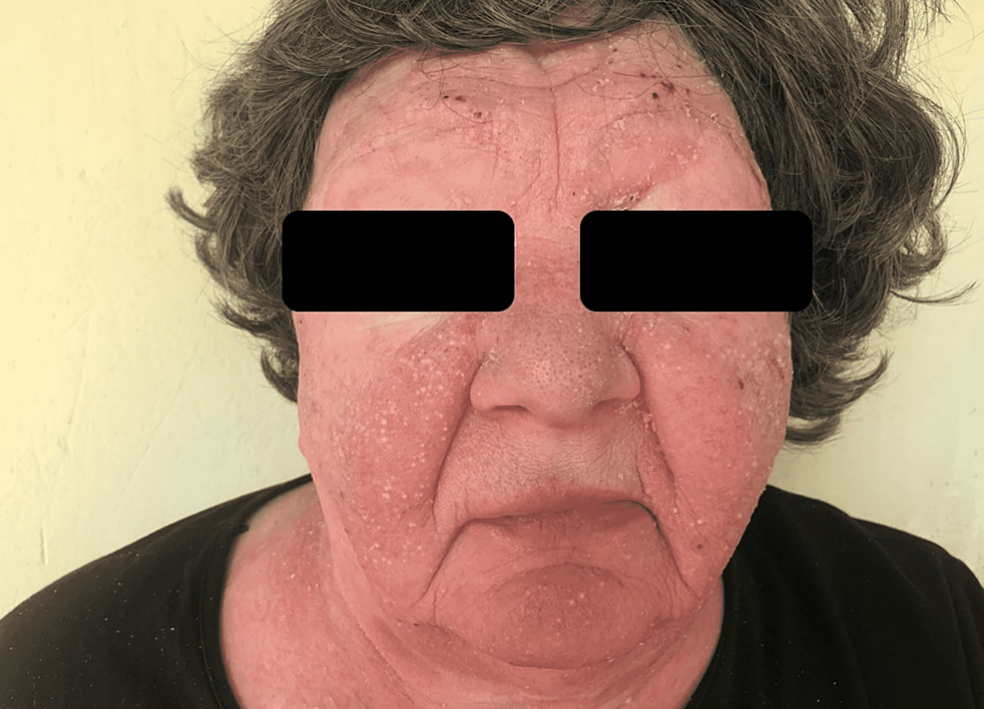
Erythematous-squamous plaques and pustules

A 69-year-old female without significant medical history or exposure to radiation or toxic chemicals, presented in our dermatology clinic in 2018 with scaly, indurated, and itchy erythematous-violaceus patches and plaques, some of them with a tendency to tumor transformation, disseminated at the entire body surface (Figure 1). In addition, erythematous-squamous plaques and pustules on the face were seen (Figure 2).

**Figure 3:**
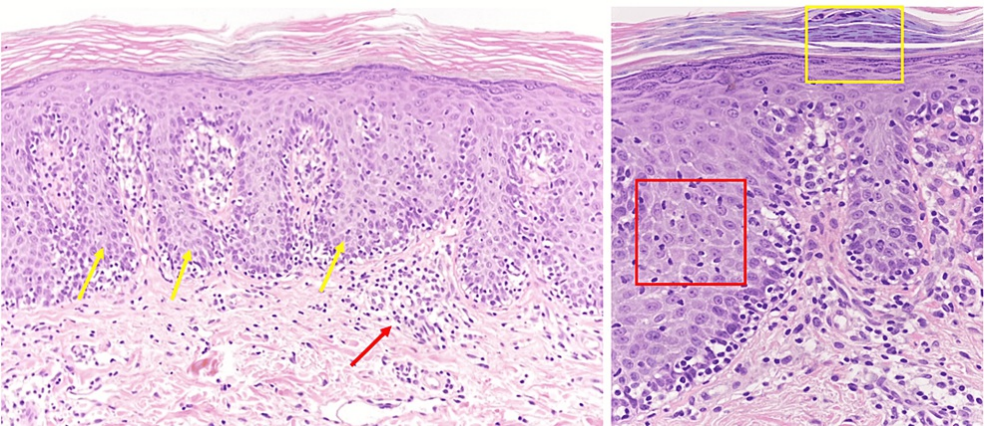
Photomicrograph (x100, x400, hematoxylin and eosin stained) Image showing epidermis with irregular hyperplasia (yellow arrows) , parakeratosis (yellow square), superficial and deep dermal infiltrates perivascular and interstitial; epidermotropism (red square); nodular lymphoid aggregates located in the deep dermis (red arrow)

**Figure 4:**
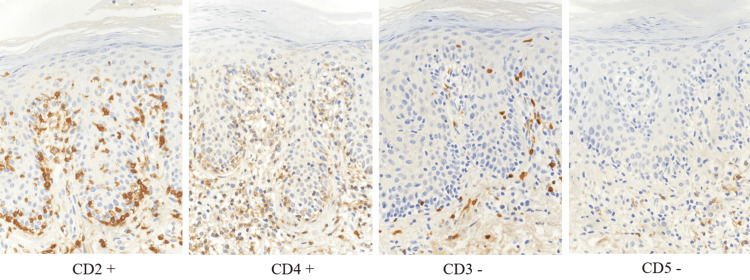
Immunohistochemical staining Image revealing positivity for CD2 and CD4 and negative results for CD3 and CD5 markers

The patient reported that the first lesions appeared in 2009 on the trunk, as small erythematous patches. In 2017, the patient presented to another dermatology clinic for erythematous-squamous pruriginous plaques on the trunk and diffuse alopecia. The patient stated that a skin biopsy was performed at that time, with a histopathological exam consistent with mycosis fungoides. Systemic therapy with antihistamines and retinoids, narrowband UVB phototherapy, and topical therapy was instituted, resulting in moderate relief of lesions. In 2018, during the hospitalization in our clinic, two skin biopsies were performed, considering the absence of medical records of the patient. The histopathological examination revealed: epidermis with irregular hyperplasia, parakeratosis, superficial and deep dermal infiltrates perivascular and interstitial, epidermotropism with lymphocytes with irregular nuclear contour and vacuolated cytoplasm, aligned at the level of the basal layer of the epidermis, nodular lymphoid aggregates located in the deep dermis (Figure 3), typical changes for mycosis fungoides. Histopathology was completed with immunohistochemical tests. The immunophenotypic profile revealed positivity for CD2, CD4, CD7 associated with negative markers CD3, CD5, CD8, CD20, supporting the diagnosis of mycosis fungoides (Figure 4).

In addition, blood tests revealed inflammatory syndrome (erythrocyte sedimentation rate (ESR) 32 mm/h), leukocytosis with neutrophilia, and elevated lactate dehydrogenase (LDH) (701 U/L). Normal values of ionogram parameters and negative viral screening, including anti-human T-cell leukemia virus type 1 (HTLV-1) antibodies, were seen. The computed tomography showed no pathological lymphadenopathy but only an adenoma of the left adrenal gland. Considering the clinical aspect of the lesions correlated with the laboratory tests and imaging: histopathological and immunohistochemical examination, leukocytosis with neutrophilia, lack of lymphadenopathy, we excluded Sezary syndrome, and we confirmed the diagnosis of mycosis fungoides, placing the patient in a stage IIB of disease (T3N0M0B0). Systemic antibiotic therapy was initiated, considering the increased risk of bacterial superinfection of the lesions due to skin barrier disruption and depressed local and systemic immune response to various pathogens. The patient also received systemic corticosteroids, phototherapy, topical emollients, and potent topical corticosteroids. She was discharged with the recommendations for systemic treatment with acitretin 10 mg/day, oral corticosteroids with progressive tapering doses, narrowband UVB phototherapy, and radiotherapy appointment to initiate superficial external radiotherapy. We also considered a hematological hospitalization necessary for further investigations and appreciation of the opportunity to start systemic chemotherapy. In 2019, the patient returned to the clinic with fatigue, large and ulcerated skin ulcers, disseminated in the hands, abdomen, and scalp, with a tendency to erythroderma, suggesting progression to stage III of the disease. The patient presented to the hematology department, where an osteomedullary biopsy was performed that did not show bone marrow involvement, failing to meet the criteria for initiating systemic chemotherapy. Given the evolution of the disease under treatment, we decided to increase the dose of acitretin to 30 mg/day, associate psoralen with UVA (PUVA) phototherapy, topical treatment with 5-fluorouracil 5% on skin tumors, and topical corticosteroids on the placards. In addition, targeted anti-lymphocyte T systemic treatment is recommended, considering the rapid evolution of the skin lesions (within one year) under conventional therapy for mycosis fungoides. Considering the clinical and paraclinical data, hematologists decided to initiate EPOCH systemic chemotherapy (etoposide, prednisone, vincristine, cyclophosphamide, doxorubicin), causing the improvement of the lesions. However, after the third dose, the patient died due to respiratory failure.

## Discussion

Mycosis fungoides accounts for less than 1% of non-Hodgkin's lymphomas but is the most common cutaneous subtype of these conditions. According to the WHO-EORTC (World Health Organization-European Organization for Research and Treatment of Cancer) classification, based on phenotypic and molecular characteristics, primary skin lymphomas are classified as cutaneous lymphomas with T lymphocytes, B lymphocytes, natural killer (NK) cells, and unclassified primary skin lymphomas [6]. Etiopathogenic, mycosis fungoides is characterized by the proliferation of lymphocytes that express the T-cell receptor (CD4+) on the surface, causing increased production of Th2-type cytokines, with indolent evolution and favorable prognosis when diagnosed in the early stages of the disease [3]. TNMB (Tumor, Nodes, Metastasis, Blood) is the classification system for mycosis fungoides developed by the International Society for Cutaneous Lymphomas (ISCL) and the European Organization for Research and Treatment of Cancer (EORTC) in which are evaluated: skin (T1-T4), lymph nodes (N0-N3), viscera (M0-M1), and peripheral blood (B0-B1), determining the stage of the disease (Table 1) [7].

**Table 1:** TNM classification and clinical staging of mycosis fungoides (adapted from NCCN clinical practice guidelines, 2020) BSA: body surface area; NCCN: National Comprehensive Cancer Network; TNM: Tumor, Nodes, Metastasis

Stage	T	N	M	Description
IA	T1	N0	M0	<10% BSA patch/plaque
IB	T2	N0	M0	>10% BSA patch/plaque
IIA	T1-T2	N1	M0	Patch/plaque with palpable nodes without histological involvement
IIB	T3	N0-N1	M0	Cutaneous tumors with/without palpable nodes
III	T4	N0-N1	M0	Erythroderma with/without palpable nodes
IVA	T1-T4	N2-N3	M0	Non-palpable or palpable nodes with histological involvement
IVB	T1-T4	N0-3	M1	Visceral involvement

The mechanisms underlying the etiopathogenesis of the disease are not fully elucidated. This condition is considered the result of the interaction between genetic and environmental factors. There are two hypotheses: first, the continuous proliferation of T lymphocytes in the skin that causes chronic inflammation, followed by the malignant transformation of a T lymphocyte clone through a persistent antigenic stimulus (chemicals, radiation, bacterial superantigens-Staphylococcus aureus). The second hypothesis is the existence of a specific viral agent such as HTLV-1, which acts as a trigger in the malignant transformation of T cells and stimulates their proliferation [8].

Although considered a rare disease, mycosis fungoides requires high clinical suspicion due to the improved prognosis in the patients with early diagnosis. In the early stage, also called premycotic, the lesions are macules and erythematous patches, slightly scaly, with variable diameters, especially on the gluteal region and the thighs. The differential diagnosis is made with inflammatory dermatoses, but the central pathology that causes diagnostic difficulties is parapsoriasis in plaques, considered by some authors an initial phase of mycosis fungoides [5]. In contrast with parapsoriasis in small plaques, parapsoriasis in large plaques clinically presents larger plaques with atrophy and histopathological changes similar to those in mycosis fungoides, which is why some authors consider it an early form of mycosis fungoides [9]. In the plaque stage, known as mycotic, the lesions are erythematous-violaceus, palpable, infiltrated, with well-defined edges and polycyclic appearance. The lesions have a papular or nodular appearance in the tumor stage, sometimes ulcerating, and may coexist with earlier stages [1].

The extent of cutaneous and extracutaneous manifestations influence the prognosis of the disease. In the initial stage of the disease, with limited patches or plaques affecting less than 10% of the skin surface, the prognosis is excellent with a survival similar to the general population. At the stage of generalized patches/plaques extending to more than 10% of the skin surface, the average survival is about 11 years. In the stage of skin tumors (IIB) or erythroderma (III), the average survival from diagnosis decreases to 3 years and 4.5 years, respectively [10]. Thus, there are data in the literature according to which patients in the tumor stage (T3) and erythrodermic stage (T4) share the same prognosis, with similar survival rates. However, there are also conflicting data showing that patients in the T3 stage have a more unfavorable prognosis than those in the T4 stage, contradicting the current classification system [11,12]. The most important prognostic factors are the stage of skin lesions (T), noting that generalized tumors are associated with a worse prognosis than solitary and extracutaneous involvement (lymph nodes, visceral metastases, peripheral blood) [13]. Other prognostic factors are the patient’s age (<65 years or> 65 years), disease stage, peripheral blood eosinophilia, serum beta2-microglobulin level, and serum lactate dehydrogenase [14]. LDH is one of the hematological markers that significantly influence the prognosis of malignancies. A high serum level is an independent factor of unfavorable prognosis [13,15]. LDH is a cytoplasmic enzyme that facilitates the process of glycolysis by converting pyruvate to lactate. Elevated serum LDH levels are associated with accelerated glycolysis, characteristic of malignant cell metabolism. An acidic microenvironment caused by lactic acid accumulation can cause the development of local tumor vascularization by stimulating vascular endothelial cell growth factor (VEGF) and interleukin-8 (IL-8)-mediated angiogenesis, increased tumor cell proliferation associated with decreased apoptosis, cell invasion, and metastasis [16]. LDH is an unfavorable prognostic marker, but more studies are needed to evaluate its involvement in disease activity and progression.

Currently, there is no cure for mycosis fungoides except for allogeneic stem cell transplantation. The goal of treatment is to increase the patient’s overall survival and quality of life by maintaining the remission of the disease. Mycosis fungoides is an indolent disease, and current diagnostic and treatment guidelines (NCCN, EORTC, European Society for Medical Oncology (ESMO)) recommend a multidisciplinary, step-by-step approach adapted to the stage of the disease, taking into account the prognostic factors of each patient. Topical therapies and targeted radiotherapy are used for the early stages of the disease, and the progression determines the need for supplementation with systemic therapies [17].

This clinical case presents a natural history of the disease, with the onset of erythematous patches and plaques with a nonspecific appearance, in the non-photo exposed skin areas, with indolent evolution for nine years. The diagnosis of mycosis fungoides was established in the stage of plaques and tumors, followed by rapid progression with death after one year. The risk of rapid disease progression could have been anticipated by the following prognostic factors: advanced age (69 years), stage of skin lesions (stage T3 with a tendency to progress to T4), and elevated serum LDH. We performed therapeutic management according to diagnostic and treatment guidelines: topical therapy, phototherapy, and conventional systemic therapy. Initially included in stage IIB of the disease, the patient underwent topical cortisone therapy, narrowband UVB phototherapy, associated with systemic antibiotic treatment, high-dose corticosteroids, and oral retinoids (acitretin 10 mg/day). Given the progression of the disease under conventional medication, it was necessary to combine with the pre-existing therapy, topical 5-fluorouracil 5%, and PUVA therapy. The dose of systemic retinoid was increased to 30 mg/day of acitretin with a continuous evolution of the lesions. Given the clinical and paraclinical data, hematologists decided to initiate systemic chemotherapy with the EPOCH regimen, which resulted in the patient’s death due to respiratory failure after the third cycle of treatment. In mycosis fungoides, chemotherapy is often used as second-line therapy, EPOCH chemotherapy being associated, in advanced mycosis fungoides, with a favorable response rate and safety profile [18]. Even though there are data from the literature which show its effectiveness when used first-line, the clinical response is limited, associated with rapid recurrence of the disease and the risk of adverse effects. [19]. However, data from the literature do not show a significant impact on overall survival following combination, aggressive chemotherapy compared to sequential therapy [20]. Although many diagnostic and treatment guidelines have been developed, the therapeutic management of these patients varies widely, and the low incidence of the disease is associated with a limited number of studies targeting therapy lines. In our country, due to the lack of standardized protocols regarding these patients, the therapeutic approach is often made according to the clinician’s experience.

## Conclusions

Mycosis fungoides is a rare disease, with an increasing incidence and natural evolution with worsening skin lesions and systemic involvement in the absence of treatment. Characterized by heterogeneity, mycosis fungoides requires a high level of diagnostic suspicion because early diagnosis made by biopsy supplemented with immunohistochemistry results in an excellent prognosis. However, in most cases, the diagnosis of the disease remains ambiguous for a long time because it can mimic many skin disorders, patients experiencing itchy rashes with inconclusive biopsies, impossible to manage therapeutically. This clinical case highlights the importance of early diagnosis of mycosis fungoides, if possible, by repeated minimally invasive methods. These cases must have a multidisciplinary approach (dermatologist, oncologist, hematologist) to achieve a personalized and standardized treatment from the early stages of the disease, given the invasive nature and the unfavorable prognosis with the evolution of the disease.
